# Georgia’s Cancer Awareness and Education Campaign: Combining Public Health Models and Private Sector Communications Strategies

**Published:** 2004-06-15

**Authors:** Demetrius M Parker

**Affiliations:** Georgia Department of Human Resources, Division of Public Health

## Abstract

The Georgia Cancer Awareness and Education Campaign was launched in September 2002 with the goals of supporting cancer prevention and early detection efforts, heightening awareness of and understanding about the five leading cancers among Georgia residents, and enhancing awareness and education about the importance of proper nutrition, exercise, and healthy lifestyles. The inaugural year of the campaign is outlined, beginning with adherence to the public health principles of surveillance, risk factor identification, intervention evaluation, and implementation. A strategic and integrated communications campaign, using tactics such as paid advertising, public service announcements, local community relations, media releases, a documentary film, special events, and other components, is described in detail with links to multimedia samples. With an estimated budget of $3.1 million, the first year of the campaign focuses on breast and cervical cancer screening and early detection.

## Introduction

*Media Advocacy and Public Health:* Power for Prevention is perhaps my new favorite communications resource (1). In the foreword, Michael Pertschuk compliments the authors for reaching beyond frustration with the media’s behavior, which is described as generally “unresponsive to democratic needs” (1). The authors move toward remedies by discussing strategies that encourage collaboration with the media to serve the community’s interests in social justice and especially public health.

Georgia’s Cancer Awareness and Education Campaign (CAEC) put those kinds of strategies into practice by teaming with the local media and a cadre of community-based organizations to focus on the need for intervention to reduce the burden of cancer in Georgia. In September 2002, private sector leaders, the Governor’s Office, the Georgia Cancer Coalition, and the Georgia Department of Human Resources, Division of Public Health (DHR, Division of Public Health) launched the CAEC, a statewide, community-inclusive, population-based cancer intervention campaign. Our goals are to support cancer prevention and early detection efforts, to heighten awareness of and understanding about the five leading cancers among Georgia residents, and to enhance awareness and education about the importance of proper nutrition, exercise, and healthy lifestyles.

While we planned to expand our efforts to include colorectal, prostate, and skin cancers in future years, we selected breast and cervical cancer screening and early detection as the focus of the campaign’s inaugural year (September 2002 through June 2003) primarily because efforts to combat these two diseases are supported by a highly developed public health infrastructure within Georgia. Our budget for the first year of the program was approximately $3.1 million.

The CAEC uses strategic and integrated communications efforts enabled by a comprehensive collaboration of fully vested individuals and organizations with the capacity to effect positive behavioral change in Georgia’s cancer control efforts. The architects and stakeholders of the CAEC include the public health system, epidemiologists, physicians, nurses, health promotion and health educators, communications experts, community-based cancer organizations, and volunteers, including cancer survivors. The CAEC network also includes hospitals, national cancer-focused organizations, and federal, state, county, and local government officials. The CAEC also includes the media as a major partner.

## Planning and Implementing the Campaign

How did we build the campaign? How has Georgia’s media responded to this ambitious effort? This article outlines the answers to these questions, starting with the campaign’s immersion in public health principles (2).

### Surveillance research

The CAEC is science based, constructed according to a traditional public health model. Beginning with surveillance, the first step was to call upon the epidemiology department of the DHR, Division of Public Health. The CAEC extracted data from the Georgia Behavioral Risk Factor Surveillance System 2000 (3) and the Georgia Cancer Data Report 2000 (4). This is part of what we learned:

- In Georgia, cancer is the second leading cause of death, accounting for one in four deaths annually (4).
- One out of every two men and one out of every three women in Georgia will develop cancer during their lifetime, unless current trends are reversed (4).
- Breast cancer is the second leading cause of cancer death among Georgia females (4).
- The vast majority of cervical cancers can be prevented (4).

The data gave demographic, geographic, and cultural information that allowed us to develop the specific content of the survey tools — qualitative focus groups and quantitative surveys — that would enable us to create the most appropriate and influential media messages, images, and community outreach to accomplish the goal of the CAEC. The data also provided baseline information necessary for measuring campaign effectiveness.

### Risk factor identification

With these and other data, the CAEC moved on to the second step of the public health model — risk factor identification. The goal was to determine attitudes and beliefs about cancer screening and the themes and messages most likely to motivate Georgians to be checked more frequently for cancer. We used this research to develop motivational and informational messages to be used throughout Georgia in a multi-tactical, culturally inclusive communications campaign.

We began with qualitative focus groups. In September 2002, seven two-hour focus groups (six English, one Spanish) were conducted in geographically diverse parts of the state (i.e., southeast, southwest, and northeast Georgia in addition to the metropolitan Atlanta area). Women aged 40–75 provided their views on cancer-awareness issues. We also tested several possible campaign messages. Key findings from the focus groups included the following:

- Women generally know it is important to be screened.
- If they are not screened, it is because they do not have insurance or cannot afford it, or their physicians did not tell them they need to be screened.
- Women have little awareness of programs available from public health departments.

In October 2002, we conducted a benchmark survey to determine knowledge, attitudes, and behaviors of Georgians aged 45–74 regarding screening for five key cancers: cervical, breast, prostate, colorectal, and skin. A professional telephone survey firm randomly contacted 27,000 Georgians to achieve a final sample size of 1002 respondents. The group consisted of 51% women and 49% men. Key findings from the phone survey included the following:

- The number-one reason people obtain cancer screenings is “I don’t want to get cancer.” Thus, screening is generally viewed as a preventive measure.
- The number-one reason people do not obtain regular screenings is somewhat vague and nonspecific: “No special reason. I just don’t.”
- The second most frequently cited reason for not obtaining screening is “I don’t think I’m high-risk for that.”
- The third most mentioned reason for not obtaining screening is “lack of health insurance” and “no money to pay” for out-of-pocket screening.
- The number-one way people get health information is from their doctor.
- The second primary method people receive health information is from news on television and in newspapers and magazines.

### Evaluation of messages

Campaign MaterialsTake a tour of selected print materials from Georgia’s Cancer Awareness and Education

**Figure:**
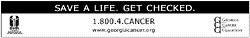
Tagline Identity Visual: "Save a life. Get Checked" logo with the cancer 1-800 number and the Georgia Department of Human Resources and Georgia Cancer Coalition logos Headline Copy: "Save a life. Get Checked." Copy: 1.800.4.CANCER www.georgiacancer.org Visual: Georgia Department of Human Resources (LOGO) Georgia Cancer Coalition (LOGO)

**Figure:**
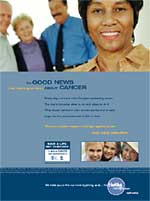
Georgia Expo Ad Visual: African American woman smiling in front, a group of people in the back; type in the middle with bright colors Headline Copy: The Good News about Cancer Copy: Every day, more and more Georgians are beating cancer. The key is knowing what to do and when to do it. When breast, cervical or colon cancers are found at an early stage, the five-year survival rate is 90% or more. There is no better weapon in the fight against cancer than early detection. Visual: Save a life. Get checked. 1.800.4.CANCER www.georgiacancer.org (LOGO) Georgia Department of Human Resources (LOGO) Georgia Cancer Coalition (LOGO) The Healthy Georgia Expo (LOGO) At bottom: other women "3 generations" representing a family

**Figure:**
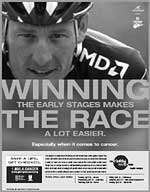
Tour Ad Visual: Lance Armstrong’s face on top left with racing gear on. Headline big and bold underneath. Headline Copy: Winning the early stages makes the race a lot easier. Especially when it comes to cancer. Copy: The manner in which Lance Armstrong won his tremendous battle against cancer was heroic. But he’ll be the first to tell you, it would have been a lot easier if he had caught it early. So when you come to watch Lance and all the other cyclist’s in this year’s Dodge Tour de Georgia, be sure to visit the Healthy Georgia Expo at any of the six finish cities. You’ll learn about screening tests used to detect cancer early, as well as information on how to live a healthy lifestyle and a number of other great ways to fight cancer. Finish cities and dates: Tuesday, April 20- Macon; Wednesday, April 21-Columbus; Thursday, April 22-Rome; Friday, April 23-Dahlonega; Saturday, April 24- Hiawassee/Young Harris; Sunday, April 25- Alpharetta Visual: Save a life. Get checked. 1.800.4.CANCER www.georgiacancer.org (LOGO) Georgia Department of Human Resources (LOGO) Georgia Cancer Coalition (LOGO) The Healthy Georgia Expo (LOGO)

**Figure:**
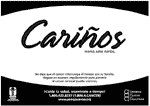
Cariños Visual: “Cariños” in large print with other type below. The 1800 number and website are at the bottom. Headline Copy: Cariños: Mamá sabe darlos. (Affection: Mom knows how to give it.) Copy: No deje que el cancer interrumpa el tiempo con su familia. Hágase un examen regularmente para prevenir el cáncer cervical (cuello uterino). (English Translation) Do not allow cancer to interrupt the time with your family. Get a regular exam to prevent cervical cancer (uterine neck). (Take care of your health, get examined regularly) Visual: Save a life. Get checked. 1.800.4.CANCER www.georgiacancer.org (LOGO) Georgia Department of Human Resources (LOGO) Georgia Cancer Coalition (LOGO)

**Figure:**
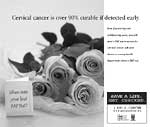
Roses Visual: A bouquet of roses with a card asking “When was your last PAP test?” and information about getting a PAP test and asking your doctor or county health department about a PAP test. Headline Copy: Cervical cancer is over 90% curable if detected early. Copy: Even if you’re beyond childbearing years, you still need a PAP test to screen for cervical cancer. Ask your doctor or county health department about a PAP test. Visual: Save a life. Get checked. 1.800.4.CANCER www.georgiacancer.org (LOGO) Georgia Department of Human Resources (LOGO) Georgia Cancer Coalition (LOGO)

**Figure:**
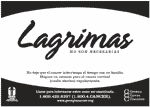
Lagrimas Visual: “Lagrimas..no son necesarias” in large print. Type underneath warning to not let cancer interrupt your time with your family by getting checked for cervical cancer regularly. Headline Copy: Lagrimas: No son necesarias. (Translation: TEARS are not necessary) Copy: No deje que el cancer interrumpa el tiempo con su familia. Hágase un examen regularmente para prevenir el cáncer cervical (cuello uterino) (English Translation) Do not allow cancer to interrupt the time with your family. Get a regular exam to prevent cervical cancer (uterine neck). Llame pare informarse sobre donde puede hacerse la prueba. 1.800.422.6237 (1.800.4.CANCER). www.georgiacancer.org (English translation) Call for more information on where you can get your exam. Visual: 1.800.4.CANCER / www.georgiacancer.org (LOGO) Georgia Department of Human Resources (LOGO) Georgia Cancer Coalition (LOGO)

Grounded in Georgia-specific cancer data, opinions, and lifestyles, we advanced to the third step of the public health model — intervention evaluation. How can we ensure that the communications intervention strategies we develop will work? We worked with our communications agencies to develop a plan that would reach the full potential of public health theory and science.

Based on our research findings, including initial focus-group feedback on potential tag lines, we developed messages in English and in Spanish designed to motivate Georgians to “take action now to get checked for cancers.” Our strategy was to make certain that the core information, guidance, and messaging was consistent across all modes of communication, including television, radio, print, Internet, public presentations, reports and brochures, and our documentary film, *An Important Conversation — Georgia Speaks*. We sought qualitative feedback on our messages from key public health and cancer-care professionals. Feedback revealed that the message most influential in motivating individuals to get checked for cancer or to obtain more information about the disease was “Save a life. Get checked.” Feedback also indicated that the message works because it is not an order or command to do something. Instead, it is perceived as personal, suggestive, simple, and memorable. For campaign planners, the message is flexible and easily applicable to all forms of communication — from advertising to speeches to brochures.

### Implementation

With confirmation that our target audiences would embrace our key message and that we could apply it across all communication methods, we moved forward with the fourth step in the public health approach — implementation. What kinds of tactics did we use to implement the CAEC? We executed the campaign through five communications tactics: 1) media releases; 2) public service announcements (PSAs); 3) a statewide media buy; 4) a short documentary film; and 5) grassroots community-outreach efforts fulfilled by statewide partnerships. (The CAEC Resource Site is available at cancer.fmeclients.com.)

When the CAEC was launched, our goal was to place a television and radio ad by the following October during National Breast Cancer Awareness Month. Because we were only in the planning stages of producing our own advertising during September and October, we conducted a national search to find the best pre-produced PSA. With the support of our public relations agency, we acquired a 30-second television PSA from the California Department of Health Services. The PSA features internationally renowned writer and actor Maya Angelou, and it also has a radio version. We received permission to retag the PSA with the toll-free number of our campaign partner, the National Cancer Institute’s Cancer Information Service (1-800-4CANCER). We also included the logos of the Georgia Cancer Coalition and DHR, Division of Public Health. The toll-free service had been developed in partnership with the National Cancer Institute’s Cancer Information Service. We branded all CAEC communications efforts with these logos and the toll-free number.

Campaign Materials

**Figure:**
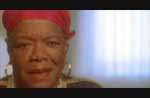
"Maya" Watch "Maya" PSA

Dr. Maya Angelou As we get older, our chances of developing breast cancer increase.
(Couple dancing.)
Graphics WANNA treated her breast cancer in 1979.
Dr. Angelou However, every year, more women continue to live meaningful lives because their breast cancer was discovered and treated early.
Graphics Yuk Hing treated her breast cancer in 1996.
Dr. Angelou Every woman deserves to be healthy.
Graphics CARMEN treated her breast cancer in 1998.
Dr. Angelou And a good way to make sure you don't have breast cancer is to have a mammogram every year. If you're over 40, call about a free mammogram. Do it for yourself and the people you love Every woman counts, every year.
Graphics GEORGIA CANCER COALITION 1-800-4CANCER (422-6237) www.georgiacancer.org (End.)

**Figure:**
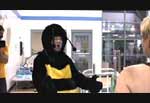
"Bee" Watch "Bee" PSA

A WOMAN IS LEADING AN ELDERLY GROUP IN A WATER-AEROBICS CLASS.

A MAN DRESSED AS A BEE COMES IN AND BEGINS DELIVERING A SINGING TELEGRAM.
FEMALE VOICE All right, two more laps. And two. Arms good and tired, yeah? (Laughter.)
BEE CHARACTER Bzzzzz. A message from Allison: (Sings.) "Be smart, be brave. What I'm really trying to say, someone needs to take a peak at a part of which we rarely speak." (End singing.) Oh, what the heck. Just get your darn colon checked, okay! (Laughter.)
BEE CHARACTER Okay. That's the buzz. I'm out of here, people. You were great. Even you. Thank you.
GRAPHICS GEORGIA CANCER COALITION 1-800-4CANCER (422-6237) www.georgiacancer.org (End.)

**Figure:**
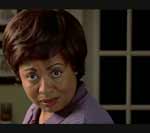
"Oh, Harold" Watch "Oh, Harold" PSA

**SFX:** MUSIC UP AND UNDER.

A MAN IS SEATED AT A DINING ROOM TABLE READING THE NEWSPAPER. OVER HIS SHOULDER, WE CAN SEE HIS WIFE IN THE KITCHEN. WITHOUT LOOKING UP, HE CARRIES ON A CONVERSATION WITH HER.
HAROLD And another thing, when was the last time you had a mammogram? GO TO CLOSEUP OF ANNA.
ANNA Oh Harold, that’s sweet. Are you trying to tell me that you love me?
HAROLD No. I just heard, you know, you should get a mammogram every year. GO TO CLOSEUP OF ANNA.ANNA Oh, Harold, now you know you trying to tell me that you can't live without me. GO TO CLOSEUP OF HAROLD.
HAROLD I wouldn’t read too much into it. I’m just saying, you know, who’ll water the plants?
ANNA Oh Harold, you are a sweetheart.
VOICEOVER To find out about low- or no-cost mammograms in your area, call 1-800-4CANCER.
GRAPHICS Save a Life, Get Checked 1-800-4CANCER GEORGIA CANCER COALITION www.georgiacancer.org (End.)

**Figure:**
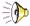
"Maya Radio" Hear "Maya" radio spot

FEMALE VOICE (Singing) "I'm every woman, so all you women, every year, go all you women. Every woman counts."
VOICEOVER Dr. Maya Angelou.
DR. MAYA ANGELOU As we get older, our chances of developing breast cancer increase. However, every year, more women continue to live meaningful lives because their breast cancer was discovered and treated early. Every woman deserves to be healthy. And a good way to make sure you don't have breast cancer is to have a mammogram every year. If you're over 40, call about a free mammogram. Do it for yourself and the people you love Every woman counts, every year.
VOICEOVER Call 1-800-4CANCER. That's 1-800-422-6237. Because...
FEMALE VOICE (Singing) "Every woman counts, every year."
VOICEOVER Brought to you by the Georgia Department of Human Resources and the Georgia Cancer Coalition.
(End.)

**Figure:**
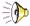
"Bee Radio" Hear "Bee" radio spot

VOICEOVER Introducing a new tool in the fight against colorectal cancer. (Buzzing.)
VOICEOVER Georgia has enlisted a swarm of bees to help us take the sting out of cancer.
BEE CHARACTER (Singing.) "Be smart, be brave, what I'm really trying to say is someone needs to take a peak at a part of which we rarely speak." (End singing.) Oh, what the heck. Just get your darn colon checked, okay!
VOICEOVER If you're over 50, you're at risk. But there's great news. Colorectal cancer is 90 percent curable if detected early. So go in and get checked, because we've got to catch it early. Remember, when it wants to, a bee can be a real pest.
BEE CHARACTER Are you going to get checked?
MALE VOICE I'm going to get checked.
BEE CHARACTER Are you going to get checked?
MALE VOICE I'm going to get checked.
BEE CHARACTER Are you going to get checked?
MALE VOICE I'm going to get checked. Okay?
BEE CHARACTER Okay.
VOICEOVER Save a life. Get checked. Ask your doctor or a health care provider about getting checked for colorectal cancer. Or call 1-800-4CANCER. Or visit Georgiacancer.org for more information. Brought to you by the Georgia Cancer Coalition and the Georgia Department of Human Resources, Division of Public Health.
(End.)

The PSA campaign and media blitz began in October 2002. The media blitz consisted of publicizing National Breast Cancer Awareness Month, the launch of the CAEC, and the availability of our toll-free number. Within 10 weeks of launch, 820 women from Georgia called the toll-free number seeking guidance on referrals for mammograms and Papanicolaou (i.e., Pap) tests. In the year preceding the campaign, only 15 people called the toll-free number. This marked the campaign’s first measure of success.

Leveraging the “Maya” PSAs, the CAEC deployed a statewide paid media campaign in English and Spanish from November 2002 through June 2003. The paid media buy was implemented through more than two dozen television stations, selected cable systems, and 29 rural newspapers that served areas not saturated with television, including the northwest, northeast, southwest, and southeast corners of Georgia. In February 2003, we rolled out our “Oh, Harold” PSA, which encourages women to obtain mammograms and is based on the findings of our focus groups and telephone surveys and other feedback. (The “Bee” PSA campaign, which focuses on colorectal cancer, was launched in March 2004.) The “Maya” PSA aired more than 550,000 times from October 2002 through the end of February 2003. “Oh, Harold” ran approximately 182,000 times from February until the end of May 2003. We estimate that the total media value of these PSAs is more than $1.1 million.

How did Georgia’s media respond to the CAEC goal? Their response was outstanding and remains at the heart of CAEC’s measurable effectiveness. Two major media partners are the Cable Advertising of Metro Atlanta (CAMA) and the Georgia Press Association. Callers to the toll-free number were motivated by the television PSA aired statewide, mostly over CAMA stations from October 2003 to April 2003. The Georgia Press Association posted CAEC news media releases and print PSAs on its Web site and encouraged its statewide membership of 150 newspapers to download CAEC resources for their readers. We will continue to use this strategy as we expand CAEC activities over the next three years of the campaign. Using feedback from the original baseline study, along with feedback from the mid-point and first-year surveys, we developed original print and broadcast PSAs and paid ads that continue to be published and aired via print and broadcast media. The campaign achieved near saturation with ads and news reports placed in the state’s 150 leading newspapers, TV cable networks, the Georgia News Network of radio stations, and Georgia Public Broadcasting, Georgia’s statewide network of radio and television stations.

We also produced a 10-minute documentary film, *An Important Conversation — Georgia Speaks*, which emphasizes the need for early detection and advises viewers to be proactive and to take control of their lives by getting regular medical examinations and screenings. (Users with Windows Media Player can view a Microsoft Media version of this documentary at http://167.193.144.238/georgiaspeaks.asx. We also offer the documentary in RealPlayer format at http://167.193.144.200:8080/ramgen/georgiaspeaks/georgiaspeaks.rm.)

The documentary features Georgians of different ethnicities, geographic areas, and professions who talk about their experiences with cancer, their roles in Georgia’s fight against cancer, and their recommendations on how to prevent and treat the disease. The film also offers information on support services such as cancer screenings and treatment.

Evidence shows that survivors are the most influential spokespersons for delivering cancer prevention messages, so we asked survivors to “star” in the film. Again, they represent Georgia’s geographic and cultural diversity and illustrate that the disease is pervasive and non-discriminatory. They address three important points learned from our telephone survey research:

- Don’t be embarrassed about having cancer.
- Don’t neglect yourself . . . get help and information.
- Do talk about cancer with your friends and loved ones.

We created a special event for the premier of the film. Cancer survivors featured in the film, along with 500 other guests, were invited to the studios of Georgia Public Television (GPTV), Georgia's statewide public television network, to view the documentary as part of an evening celebration. Segments of the event were also aired on GPTV. It received extra attention and publicity through the development of a statewide-promoted community event, Breast Cancer Prevention and Awareness Day in Georgia. In October 2003, Georgia Governor Sonny Perdue proclaimed Breast Cancer Prevention and Awareness Day in Georgia to be recognized during the third Wednesday of October every year.

The CAEC directed the overall statewide public awareness and education campaign on multiple grassroots levels. These included the following:

- Partnering with local health agencies, Regional Cancer Programs of Excellence, cancer-focused organizations, hospitals, churches, synagogues, mosques, temples, and other groups and organizations in all 159 Georgia counties.
- Providing media-trained cancer survivors as spokespersons for speaking engagements at civic and business clubs.
- Working with the media in all 159 counties to position health professionals and cancer survivors on television and radio, as well as coordinating newspaper feature stories with Georgia Cancer Coalition and DHR messages.
- Partnering with several professional and amateur sports teams in Georgia to reach their broad fan bases with the message that early detection saves lives. This included partnering with the Georgia Department of Industry, Trade and Tourism’s Tour de Georgia, a statewide professional bicycle race that reached cycling fans at venues throughout the state.
- Supporting multicultural health fairs and other educational events throughout Georgia’s 19 Public Health Districts to achieve maximum visibility, publicity, and attendance.
- Educating Georgians with screening and early detection messages through a strategic media buy covering the state with CAEC messages, particularly in regions where cancer mortality rates are highest.

Research and measurement is ongoing. Quantitative surveys at the campaign’s first-year midpoint in March 2003 and at the conclusion of its first year in June 2003 measured shifts in knowledge, attitudes, and behaviors. Both surveys were conducted by the same independent research group. The March survey had a sample size of 500 and the June survey had a sample size of 1000. Both samples were demographically and geographically representative of the population aged 45–74 in Georgia.

The March survey served to benchmark advertising awareness and recorded some attitudinal shifts among participants, revealing that for each type of cancer, particularly breast and cervical cancers, more individuals stated as reasons for their screening behavior that:

- They want to make sure they don’t have cancer.
- They are in a high-risk group.
- “You are supposed to be screened.”

The baseline survey in September and October 2002 showed that many women did not get screened because they were afraid of a positive diagnosis, and many did not believe in the effectiveness of mammograms. The March 2003 survey showed that negative attitudes decreased among women who went unscreened, their confidence in cancer screening and treatment increased, and fewer viewed a diagnosis of cancer as a death sentence.

A small overall change took place in the knowledge and behaviors of Georgians aged 45–74 regarding cancer screenings between the October 2002 and March 2003 survey:

- Although the target groups (individuals who are uninsured, who are without the care of a physician, who have annual household incomes of less than $25,000, who have a high school education or less, or who are single residents) remain the most likely to skip regular mammograms, each group is less apt to forego regular mammography now than in October 2002.
- Most Georgians are aware of advertising that addresses the need for breast cancer screening, but only a small number are aware of advertising that addresses cervical cancer screening. Most do not recall the sponsor of any advertisement, including cancer-related ads. According to our June 2003 survey, there was little overall change in the awareness of cancer advertising among Georgians aged 45–74 compared with the March 2003 survey.

## Summary

The Cancer Awareness and Education Campaign achieved successful and measurable results in its inaugural year (September 2002 through June 2003). The toll-free number attached to all messages allowed us to quantify the campaign’s impact. Before the statewide initiative began in fall 2002, an average of 1.25 calls was received per month by the call center managed by the National Cancer Institute’s Cancer Information Service. After the launch of the campaign, calls represented almost every zip code in Georgia. They continued to average 300 per month and spiked to almost 600 in January 2003.

Our partnership with the Georgia Press Association targeted a potential 3 million readers. The television and radio PSAs aired more than 745,000 times during the campaign's first year — at no charge — with an total estimated media value of more than $1.1 million.

The CAEC used the power, influence, and good will of mass media to serve the greater community interest in social justice, especially the public’s health (1). In addition, the CAEC realizes the vision of the DHR, Division of Public Health to create “a Georgia with healthy people, families, and communities, where all sectors unite by pooling their assets and strengths to promote health for all” (5).
