# Supplementary figures and images for: Georgia’s Cancer Awareness and Education Campaign: Combining Public Health Models and Private Sector Communications Strategies
Source: Prev Chronic Dis. 2004 Jun 15;1(3):A09. (PMC1253474)

SAVE A LIFE. GET CHECKED.

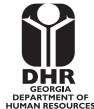

1.800.4.CANCER  
[www.georgiacancer.org](http://www.georgiacancer.org)

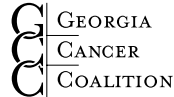

Supplement: Supplementary file 1 [file 04_0030_01.pdf]
