# Supplementary material for: Georgia’s Cancer Awareness and Education Campaign: Combining Public Health Models and Private Sector Communications Strategies
Source: Prev Chronic Dis. 2004 Jun 15;1(3):A09. (PMC1253474)
Supplement: Supplementary file 2 [file 04_0030_02.pdf]

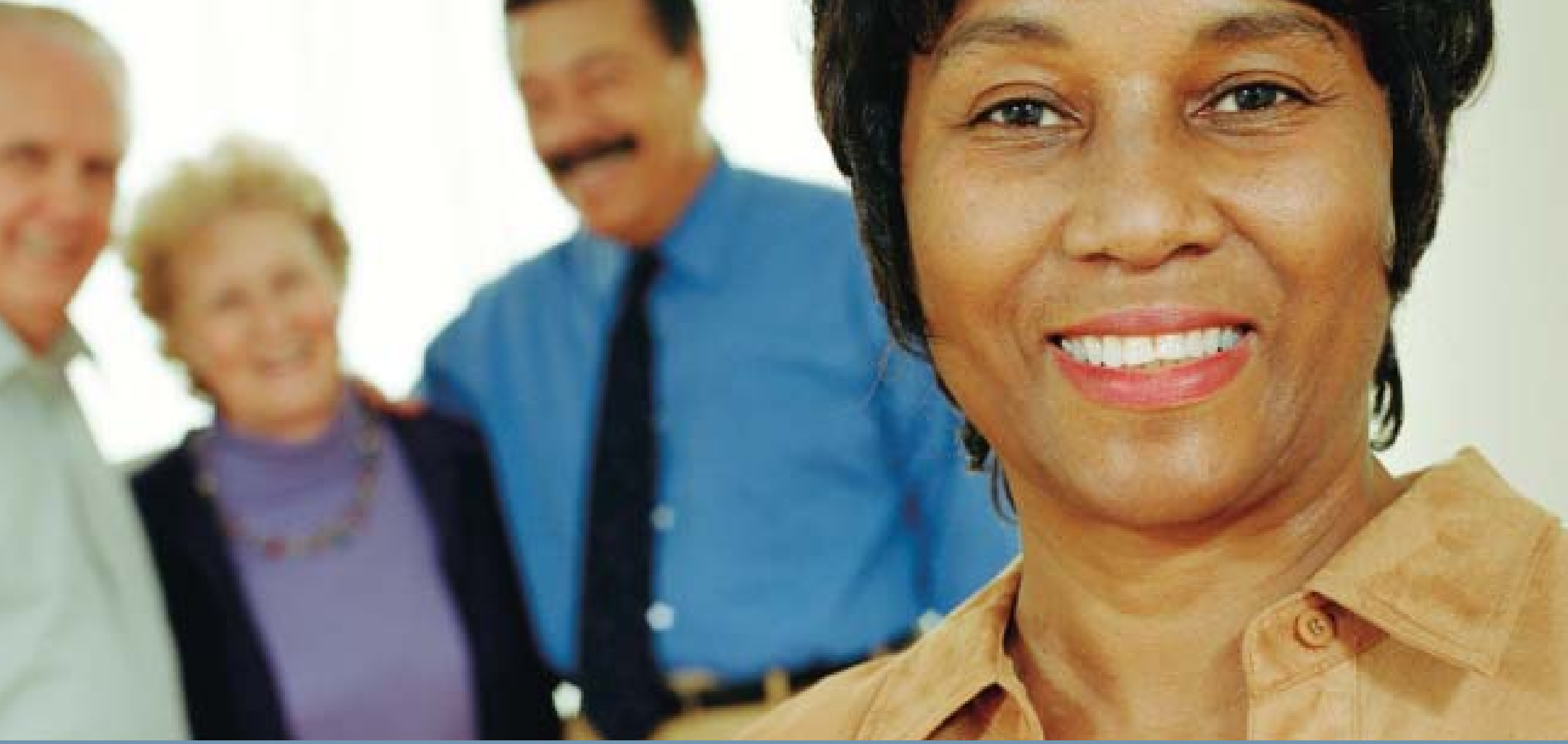

The GOOD NEWS ABOUT CANCER

(Yes, there is good news.)

Every day, more and more Georgians are beating cancer.

The key is knowing what to do and when to do it.

When breast, cervical or colon cancers are found at an early stage, the five-year survival rate is 90% or more.

There is no better weapon in the fight against cancer than early detection.

SAVE A LIFE. GET CHECKED.

1.800.4.CANCER
www.georgiacancer.org

Georgia Cancer Coalition
DHR

©2004 Georgia Department of Human Resources

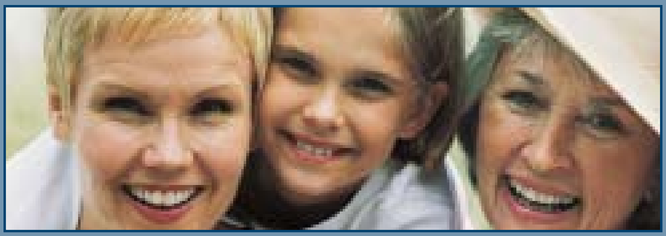

When breast, cervical or colon cancers are found at an early stage, the five-year survival rate is 90% or more. Routine screening can prevent about 15 to 30 percent of deaths from breast cancer among women over age 40. **Nearly all deaths from cervical cancer can be prevented by regular Pap tests.** Deaths from colon cancer can be reduced by at least 33% with regular screening.

We invite you to find out more by joining us at... the Healthy Georgia expo

health matters.
