# Supplementary material for: Georgia’s Cancer Awareness and Education Campaign: Combining Public Health Models and Private Sector Communications Strategies
Source: Prev Chronic Dis. 2004 Jun 15;1(3):A09. (PMC1253474)
Supplement: Supplementary file 3 [file 04_0030_03.pdf]

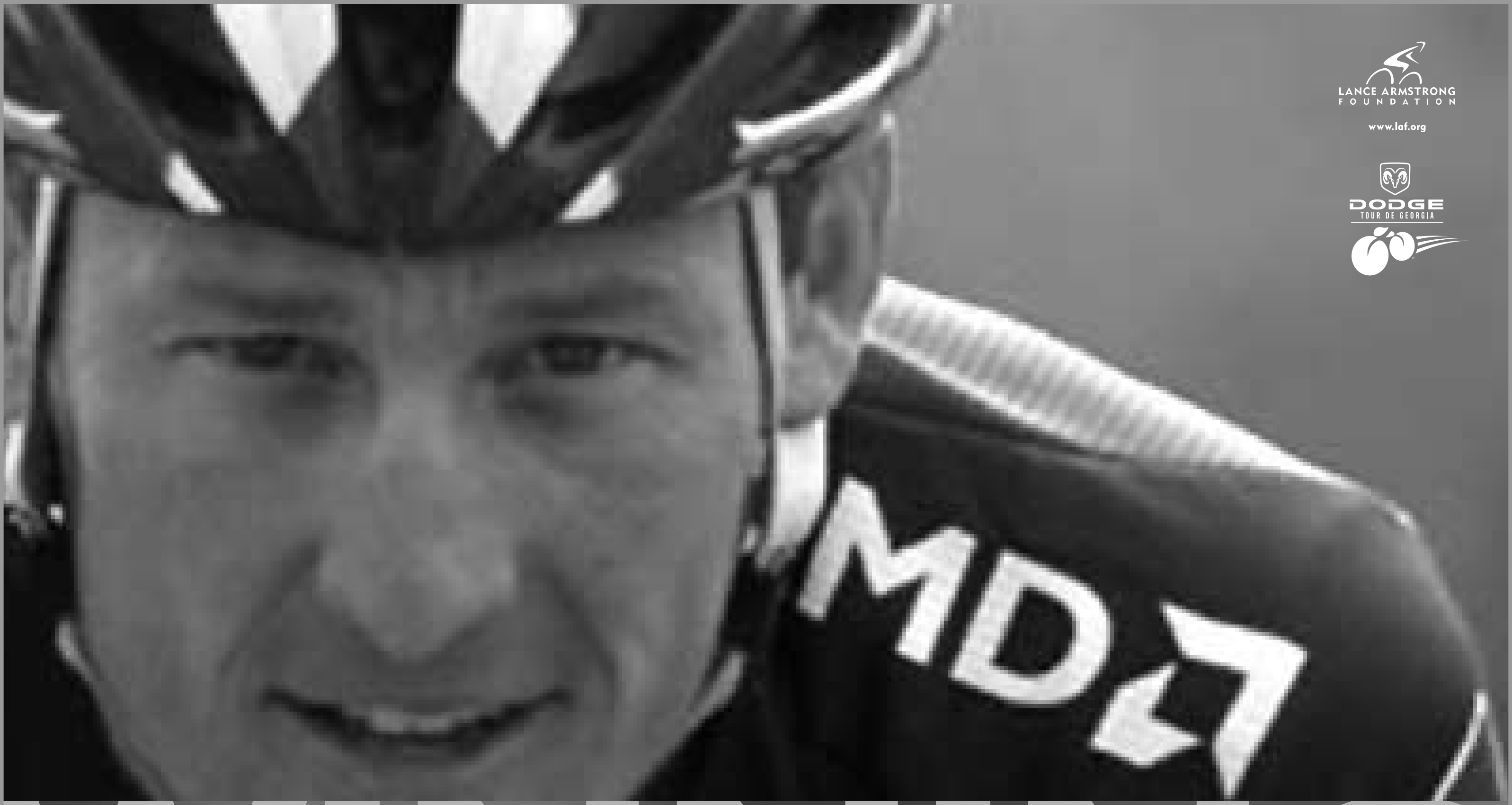

WINNING THE EARLY STAGES MAKES THE RACE A LOT EASIER.

Especially when it comes to cancer.

SAVE A LIFE. GET CHECKED.

1.800.4.CANCER
www.georgiacancer.org

Georgia Cancer Coalition

DHR Georgia Department of Human Resources

The manner in which Lance Armstong won his tremendous battle against cancer was heroic. But he'll be the first to tell you, it would have been a lot easier if he had caught it early. So when you come to watch Lance and all the other cyclists in this year's Dodge Tour de Georgia, be sure to visit the Healthy Georgia Expo at any of the six finish cities. You'll learn about screening tests used to detect cancer early, as well as information on how to live a healthy lifestyle and a number of other great ways to fight cancer.

the Healthy Georgia expo
health matters.

finish cities and dates

Tuesday, April 20 – Macon
Wednesday, April 21 – Columbus
Thursday, April 22 – Rome
Friday, April 23 – Dahlonega
Saturday, April 24 – Hiawassee/Young Harris
Sunday, April 25 - Alpharetta

©2004 Georgia Department of Human Resources
