# Supplementary material for: Georgia’s Cancer Awareness and Education Campaign: Combining Public Health Models and Private Sector Communications Strategies
Source: Prev Chronic Dis. 2004 Jun 15;1(3):A09. (PMC1253474)
Supplement: Supplementary file 4 [file 04_0030_04.pdf]

Cariños

mamá sabe darlos.

**No deje que el cáncer interrumpa el tiempo con su familia.
Hágase un examen regularmente para prevenir
el cáncer cervical (cuello uterino).**

DHR
GEORGIA
DEPARTMENT OF
HUMAN RESOURCES

**¡Cuida tu salud, examinate a tiempo!
1.800.422.6237 (1.800.4.CANCER)
www.georgiacancer.org**

Georgia
Cancer
Coalition
