# Supplementary material for: Georgia’s Cancer Awareness and Education Campaign: Combining Public Health Models and Private Sector Communications Strategies
Source: Prev Chronic Dis. 2004 Jun 15;1(3):A09. (PMC1253474)
Supplement: Supplementary file 5 [file 04_0030_05.pdf]

Cervical cancer is over 90% curable if detected early.

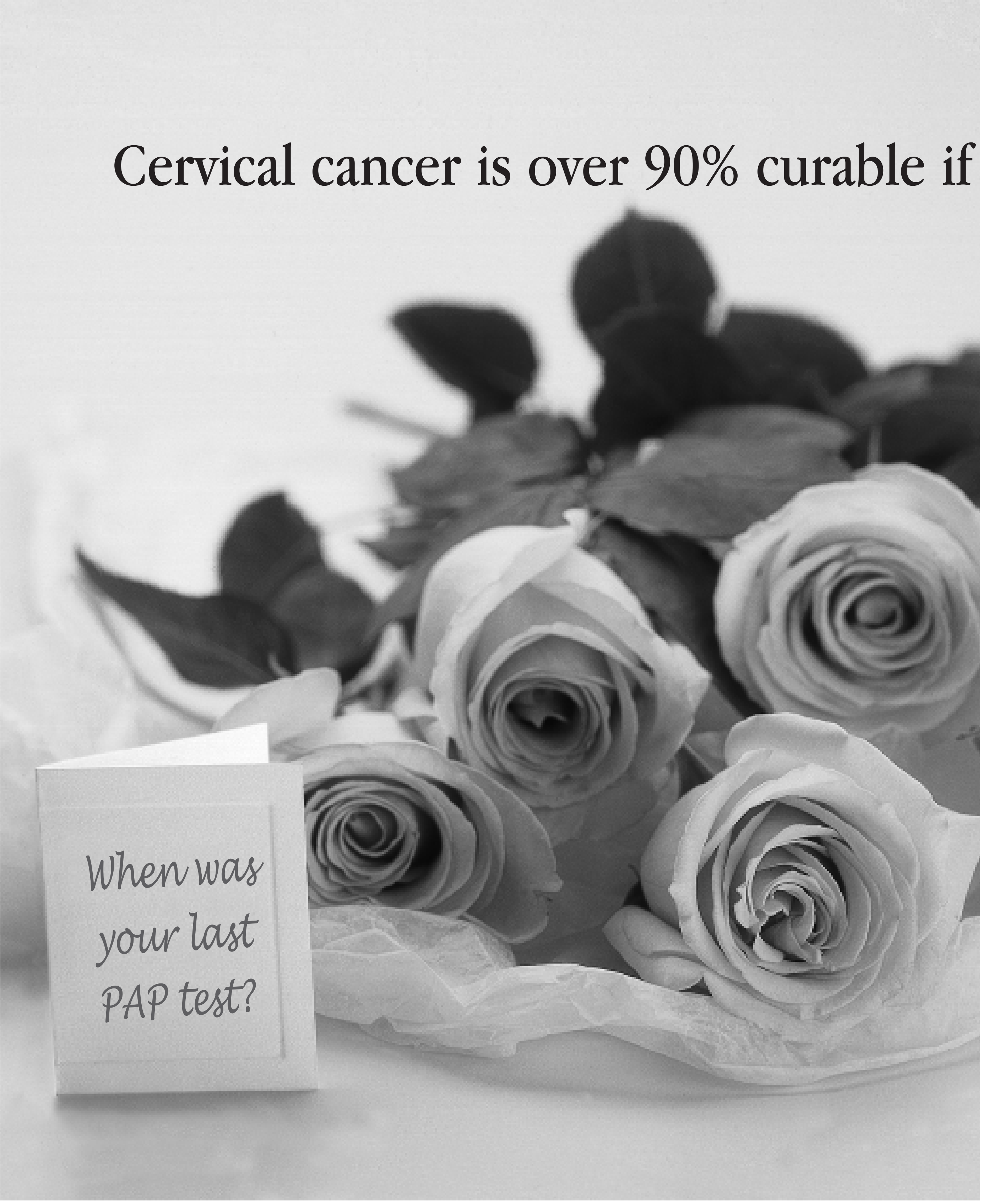

Even if you're beyond childbearing years, you still need a PAP test to screen for cervical cancer. Ask your doctor or county health department about a PAP test.

**SAVE A LIFE.
GET CHECKED.**

1.800.4.CANCER

www.georgiacancer.org

Georgia Cancer Coalition

DHR Georgia Department of Human Resources
