# Supplementary material for: Georgia’s Cancer Awareness and Education Campaign: Combining Public Health Models and Private Sector Communications Strategies
Source: Prev Chronic Dis. 2004 Jun 15;1(3):A09. (PMC1253474)
Supplement: Supplementary file 6 [file 04_0030_06.pdf]

Lagrimas

NO SON NECESARIAS

**No deje que el cancer interrumpa el tiempo con su familia.
Hágase un examen para el cancer cervical
(cuello uterino) regularmente.**

DHR
GEORGIA
DEPARTMENT OF
HUMAN RESOURCES

Llame para informarse sobre como ser examinada.

1.800.422.6237 (1.800.4.CANCER).

www.georgiacancer.org

Georgia
Cancer
Coalition
